# Evaluation Findings of a Community-Based Intervention for Older Adults With a History of Trauma

**DOI:** 10.1093/geroni/igaa057.111

**Published:** 2020-12-16

**Authors:** Carmel Rabin, Karen Edell Yoskowitz, Barbara Bedney

**Affiliations:** The Jewish Federations of North America, Washington, District of Columbia, United States

## Abstract

Between 70% and 90% of Americans aged 65 and older have experienced at least one traumatic event such as a sexual or physical assault, disaster, illness, or terrorism. Trauma exposure in older adult populations is linked to physical, mental, and cognitive decline. A new approach to improve outcomes of trauma-affected older adults is Person-Centered, Trauma-Informed (PCTI) Care, which promotes the dignity, strength, and empowerment of trauma-affected individuals by incorporating knowledge about trauma into agency programs, policies, and procedures. The Administration for Community Living/Administration on Aging has awarded The Jewish Federations of North America (JFNA) a grant to develop innovative PCTI interventions for Holocaust survivors. This includes a community-based intervention whereby local leadership councils are developed to identify Holocaust survivor needs, distribute grant funding, train caregivers in PCTI care, and forge partnerships to advance community-led Holocaust survivor care. This program has been implemented in eight major US cities where 168 community leaders dispersed 25 grants serving approximately 500 Holocaust survivors. JFNA conducted an evaluation of the first six of the eight cities to determine the impact of this community-based model on participants and Holocaust survivors and investigate the process by which a community-based model can be replicated. This evaluation used surveys and semi-structured interviews to collect data on variables including understanding of PCTI care, awareness of Holocaust survivor needs, strength of community partnerships, and leadership council sustainability. This session will review evaluation findings including best practices for community-based models of PCTI care, and applicability of findings to other older populations.

